# Pediatric Thermoregulation: Considerations in the Face of Global Climate Change

**DOI:** 10.3390/nu11092010

**Published:** 2019-08-26

**Authors:** Caroline J. Smith

**Affiliations:** Department of Health and Exercise Science, Appalachian State University, Boone, NC 28608, USA; smithcj7@appstate.edu

**Keywords:** thermoregulation, children, sweating, skin blood flow, heat stress, climate change, pollution, ultraviolet radiation, hydration, environmental stressors

## Abstract

Predicted global climate change, including rising average temperatures, increasing airborne pollution, and ultraviolet radiation exposure, presents multiple environmental stressors contributing to increased morbidity and mortality. Extreme temperatures and more frequent and severe heat events will increase the risk of heat-related illness and associated complications in vulnerable populations, including infants and children. Historically, children have been viewed to possess inferior thermoregulatory capabilities, owing to lower sweat rates and higher core temperature responses compared to adults. Accumulating evidence counters this notion, with limited child–adult differences in thermoregulation evident during mild and moderate heat exposure, with increased risk of heat illness only at environmental extremes. In the context of predicted global climate change, extreme environmental temperatures will be encountered more frequently, placing children at increased risk. Thermoregulatory and overall physiological strain in high temperatures may be further exacerbated by exposure to/presence of physiological and environmental stressors including pollution, ultraviolet radiation, obesity, diabetes, associated comorbidities, and polypharmacy that are more commonly occurring at younger ages. The aim of this review is to revisit fundamental differences in child–adult thermoregulation in the face of these multifaceted climate challenges, address emerging concerns, and emphasize risk reduction strategies for the health and performance of children in the heat.

## 1. Introduction

Globally, increased variability in environmental extremes and rising average global temperatures [1], coupled with increasing ultraviolet (UV) exposure [2] and pollution [3,4,5,6], are greatly impacting human health and performance [7,8,9]. The very limits of human thermoregulation may be tested, with multiple combined stressors of heat, dehydration, UV radiation, pollution, and noise and disease transmission all exacerbating physiological strain [10,11]. This review aims to address the topic of thermoregulation in the context of global climate challenges with emphasis placed on children, who are considered an ‘at risk’ population for increased morbidity and mortality during heat events and a range of environmental stressors.

Concepts in pediatric thermoregulation have been revisited and challenged over the past 20 years, countering the argument that children possess inferior thermoregulatory capabilities [12], particularly in mild and moderate environmental conditions. However, greater risk of adverse health events compared to healthy adults is widely recognized in more extreme environmental conditions and during physical exertion. Considering the rapidity and magnitude of predicted global climate change and adverse environmental conditions, it is prudent to understand the effects on physiological function resulting from high ambient temperatures, UV radiation [2,13,14], and pollution [4,15,16,17,18]. In addition, understanding the interplay between multiple environmental stressors and increasingly common childhood diseases and comorbidities, including obesity and the onset of preventable ‘adult’ diseases in children, is important in accurately predicting thermoregulatory responses and long-term health effects in a rapidly changing environmental landscape. This review is not a comprehensive and exhaustive review of thermoregulation in children, for which the reader is directed to other articles [19,20]. Rather, the focus of the present review will be adult–child differences in thermoregulation, with an emphasis on the adverse impact of projected environmental and climate change on thermoregulation, physiological function, overall health, and precautions for risk reduction.

## 2. Thermoregulation 

Humans regulate internal body temperature at ~37 °C via complex autonomic control of skin blood flow (SkBF) and sweating, with further local modulation [21]. Afferent inputs from central and peripheral (skin) thermoreceptors are sent to the thermoregulatory control center in the preoptic anterior hypothalamus (POAH) [22,23,24]. Inputs are integrated in the POAH before efferent sympathetic signals elicit appropriate sudomotor and vasomotor adjustments to regulate core body temperature (*T_c_*) [21]. Heat exchange and *T_c_* responses are conceptually demonstrated via the human heat balance Equation (1), with additional adjustments for respiratory heat losses:

(1) S=M−W±K±R±C−E.

Metabolic energy (*M*) is either converted into external work (*W*; negligible in most conditions) or thermal energy, which must be dissipated from the body to avoid an increase in heat storage (S). Heat exchange (both losses and gains) occurs via dry heat loss mechanisms; conduction (*K*), radiation (*R*), convection (*C*), in addition to evaporative heat loss (*E*), primarily from sweat on the skin surface but to a minor extent via the respiratory tract. Rates of heat gain and dissipation must be equivalent to maintain heat balance (S = 0) and a stable *T_c_* [25]. Under normothermic conditions, cutaneous vasomotor adjustments facilitate convective heat loss (or gain) at the skin surface to counter minor fluctuations in body temperature. During exercise and/or exposure to high ambient temperatures, heat gains exceed heat losses (S > 0), and increased *T_c_* and skin (*T_sk_*) temperatures elicit more pronounced cutaneous vasodilation and initiation of sudomotor responses [26,27,28]. SkBF can increase from ~0.25–0.30 L/min during normothermic conditions to 6–7 L/min during extreme heat exposure. To facilitate increased SkBF (and muscle blood flow during exercise in the heat), in addition to providing blood plasma as a precursor for sweat production, cardiac output (Q) increases to meet demands. Notably, when ambient temperature (*T_a_*) exceeds *T_sk_*, a reversal of the temperature gradient prevents dry heat losses from the body, and heat is gained, increasing thermal load. Evaporation of sweat is the greatest avenue of heat loss from the body during exercise and heat exposure, varying with age, exercise intensity, and hydration status. Thermoregulatory function is influenced by many factors, including cardiovascular responses, sweating rate (SR), body surface area (BSA) to mass ratio, body composition, hydration status, and nonthermal inputs. During hyperthermia, increased *T_c_* and *T_sk_* elicit thermoregulatory responses to balance heat losses and gains and stabilize *T_c_* (compensable conditions) [25]. If heat loss mechanisms are not sufficient, conditions are said to be uncompensable and *T_c_* will continue to rise, with potential progression to heat-related illness and injury, including fatal heat stroke [29].

Adult–child differences in thermoregulatory responses to warm environments are evident, largely owing to immaturity of their physiological systems, morphological, and neuroendocrine differences. Children exhibit a lower Q [30], lower whole-body SRs [31,32,33,34], and greater increases in *T_c_* during passive exercise in the heat [33,35]. Children consistently show higher SkBF responses in warm conditions compared to adults, directing a significantly greater proportion of their lower Q to the skin. Combined with significantly lower SRs, children are reported to rely more heavily on dry heat losses compared to adults [31,35]. Historically, these differences have led to the view that children possess inferior thermoregulatory responses and poorer tolerance to heat compared to adults, defined as an inability to maintain heat balance, resulting in an amplified *T_c_* response that may progress to serious heat illness without intervention [36]. This notion has been challenged [37], with accumulating evidence of compromised thermoregulatory function only at environmental extremes. This warrants further investigation with rising average global temperatures and increased frequency of heat events.

The concept of children being classified as “at risk” of heat-related illness relates to increased vulnerability to the effects of heat stress, with increased morbidity or mortality versus a healthy adult reference population [38]. Epidemiological evidence is mixed but does indicate a distinct age-related variation in heat-related morbidity and mortality [38]. Age groups at greatest risk of health-related morbidity and mortality include older adults (≥75 years) and “young children” (0–4 years) [38,39]. Death rates are lowest among children aged 5–14 years at 0.1 per million, with considerably higher infant death rates at 4.2 per million, in part owing to infants and younger children being unable to operate locks or being restrained in vehicles, in which ambient temperature is exacerbated [38]. Between 17–74 years of age, a progressive, moderate increase in heat-related deaths occurs, with considerable increases with more advanced age (≥85 years, 12.8 deaths per million). Prediction models of weather-related deaths due to excessive natural heat exposure suggest an odds ratio of 4.4 in infants (<1 year), 1.9 in young children (1–4) versus an odds ratio of 1.0 in the young adult reference population (25–34 years) [38]. Overall, the World Health Organization (WHO) predicts child mortality related to heat exposure at >100,000 deaths per year by 2050, with greatest climate change related mortality occurring in South Asia [40].

## 3. Global Climate Change 

Climate fluctuations are recognized to increase the risk and exacerbate the impact of a broad range of diseases, with extreme heat (and cold), pollution and UV exposure increasing human morbidity and mortality [41,42]. Compelling evidence from predictive models of climate change indicate with virtual certainty rising global temperatures and increasing heat wave frequency and severity, but the overall impact of climate change on human health is not fully understood [43].

Climate change will disproportionally affect certain global regions, with extreme temperatures occurring not only in traditionally warm countries, but also in regions not accustomed to high ambient temperatures. A direct temperature–mortality relationship exists and varies by geographical region and climate, with heat events causing significantly greater deaths in historically cooler areas. Predicted heat-related illness and injury could be reduced with heat acclimatization and physiological adaptation, improving the temperature–mortality relationship in cooler areas closer to that observed in warmer geographical regions [44]. In areas frequently exposed to heat, increasing frequency and severity of heat events will occur, with projected temperatures challenging the limits of human thermoregulation and acclimatization [44,45,46]. In Europe, estimates for predicted heat-related mortality in the 2080’s ranges from 60,000–165,000 in the absence of acclimatization or acclimation [44]. Notably, not only is the increase in average global land and sea temperatures important, but close attention should be given to the frequency, duration, and overall impact of heat events, during which fatalities dramatically increase [44].

Global climate change has been evident for several decades, with anticipated increases in average global temperatures of approximately 1.5 °C (2.7 °F) by 2100, with predictions ranging from 0.3 to 4.8 °C (0.5–8.6 °F) depending on varying emission scenarios predicted utilizing climate models [47,48] (pp. 1037, 1065–1068). In the United States, predictive climate models indicate increases of 1.7–6.7 °C (3–12 °F) by 2100, varying by the emission scenario. There will be an increased frequency and intensity of heat waves and related events, with altered patterns and frequency of storms (both heat- and cold-related) and precipitation [47]. Regional variation in severity of climate change will occur, with amplified arctic warming at rates ~2.2–2.4 times greater than the global average [48] (pp. 1037, 1065–1068). Predicted heat events and extremes will continue to exceed prior records, causing hundreds of thousands of additional deaths, crop destruction, and subsequent food shortages, altered disease transmission, and significantly affected economic burden [41].

Despite wide-scale scientific evidence associating greenhouse emissions and global climate change, there is limited evidence of emission reductions. The accelerated rate of increase is unparalleled in human history. Populations who are heat-sensitive or possess underdeveloped or compromised thermoregulatory responses will be most at risk of heat-related morbidity and mortality. Particular attention should be focused on the elderly, infants and children, individuals with cardiovascular, renal, metabolic diseases and related comorbidities, and individuals using medications that alter or limit mechanisms of heat dissipation. The limits of human thermal tolerance and ability to adapt to increasing global temperatures, even in healthy populations, will be tested.

## 4. Adult–Child Differences in Thermoregulation during Heat Exposure 

The American Academy of Pediatrics defines pediatrics as “the specialty of medical science concerned with the physical, mental, and social health of children from birth to young adulthood.” [49]. Discrepancies in pediatric thermoregulation literature are therefore not surprising given the large age range and developmental stages encompassed by this field. For the purposes of this review, pediatric age categories follow US Food and Drug Agency (FDA) [50] and World Health Organization (WHO) [51] guidelines for clinical pharmacology, reflecting the underlying principle of a liner relationship between weight and growth. Based on these guidelines, pediatric subpopulations are defined as (a) neonates, 0–1 month, (b) infants, 1 month to 2 years, (c) children, 2–12 years, and (d) adolescents 12–16 years. If considering adolescent, further sub-classifications based on pubertal maturational stage should be considered. Physiological responses to exercise and heat exposure differ between infants, children, adolescents, and adults owing to differences in morphological, endocrine, cardiovascular, metabolic, and thermoregulatory responses, yet guidelines are often modified from adult data. Infants and children have traditionally been considered an ‘at risk’ group with greater susceptibility to adverse physiological responses and health events during environmental extremes owing to inferior thermoregulatory capabilities [12]. Over the past twenty five years, this notion has been challenged, with limited physiological or epidemiological evidence of compromised or insufficient thermoregulatory capabilities in a wide range of environmental conditions when compared to healthy adults [19,37,52]. Many identified risk factors for heat illness in children, including hydration status, clothing, training and recovery periods, are modifiable and thus largely preventable [52]. The importance of many of these factors in pediatric thermoregulation will be reviewed, with an emphasis on recent advances and future challenges associated with climate change.

### 4.1. Morphology

Understanding alterations in thermoregulation in the context of growth and development is complex and multifaceted. Not only do children grow and mature at differing rates, but the many physical and physiological changes that impact thermoregulation during this time also occur at varied rates and times, making the pediatric population challenging to assess. Understandably, as children and adolescents grow and develop towards full maturity, adult–child differences become less pronounced. During the 1990s, several investigators suggested that thermoregulation in children differed from adults for physiological reasons beyond morphology [53,54,55], but this remains a prominent physical difference with important consequences for thermoregulation. Children are smaller than adults, with associated smaller total body surface area (BSA), lower total muscle mass, and metabolic heat production during exercise [37,56]. Notably, children have a larger BSA to mass ratio, with more effective dry heat loss and evaporative efficiency versus adults. This is advantageous in cooler and moderate conditions when *T_a_* < *T_sk_* [32,57] but also provides a greater surface area to take on heat in hot conditions. Several studies support the beneficial larger BSA:mass ratio in children during exercise in the heat [58], but this may increase the risk of heat illness as global temperatures continue to rise.

### 4.2. Body Composition

Obesity is widely recognized as a risk factor for heat-related illness and injury in both adults and children [59,60,61]. The most recent nationally representative estimates from the United States indicate that the prevalence of childhood obesity is continuing to increase: In 2015–2016, 35% of 2–19-year-olds in the National Health and Examination Survey (NHANES) were overweight (BMI ≥85th CDC percentile), and a further 24.5% obese (BMI ≥95th percentile) [62]. Globally, 50 (24–89) million girls and 70 (39–125) boys aged 5–19 years are estimated to be obese (2SD above the WHO median) [63]. In the context of the increasing incidence of overweight and obesity in children both in the USA and internationally, coupled with increasing average global temperatures, appropriate risk assessment and safety guidelines for exercise in the heat are critically important [64]. The majority of data regarding body composition and thermoregulation concerns adults, with considerable discrepancies in the literature. Several studies indicate similar responses during passive heating [65] or exercise in the heat [66], whilst others demonstrate blunted thermoregulatory (sweating) responses [67] and increased thermal strain (*T_c_*) in obese versus normal weight individuals [67]. Notably, most studies appear confounded by the effects of body mass and/or heat production introducing a systematic bias owing to study design and not adiposity per se [68]. When metabolic heat production is fixed, some of the differences are mitigated [69]. Several studies in children similarly demonstrate the physiological disadvantage of higher adiposity during exercise in the heat [70,71,72]. Higher visceral and subcutaneous fat deposits, combined with a typically lower BSA to mass ratio, may contribute to hindered heat loss in obese versus lean children [73]. By contrast, a small number of studies indicate similar heat tolerance of obese or overweight compared to normal weight children during exercise in the heat [70,74]. This has been suggested by other authors to result in part from three exercise heat acclimation sessions conducted prior to testing, which were not discussed in the studies themselves [72]. Studies incorporating exercise/heat acclimation sessions prior to experimental testing may reduce differences in thermoregulatory responses between groups but in applied terms have important implications for reducing heat illness risk in obese children. 

Importantly, total body mass is typically greater in obese compared to lean children, resulting in a greater heat production and therefore evaporative requirement during similar absolute workloads. In addition, obese children typically possess a lower maximal aerobic capacity, and therefore, when exercising at the same intensity, obese children are achieving a higher absolute workload and heat production [74]. Adipose tissue has a lower water content and lower specific heat compared to muscle (2.97 kJ·kg^−1^· °C^−1^ versus 3.66 kJ·kg^−1^· °C^−1^), with less heat necessary to increase a specific mass of adipose tissue a given temperature. It is logical to assume that both children and adults with higher adiposity may therefore experience a greater *T_c_* rise for a given heat production compared to lean individuals. Notably, the physiological impact of adiposity on thermoregulation and practical considerations for prescribing comparable workloads has recently been challenged [68]. In adults, Dervis and colleagues [68] eloquently demonstrated that during exercise in the heat, overweight or obese individuals experience a greater *T_c_* increase versus those with lower body fat, independently of differences in mass and metabolic heat production [68]. They further suggest that body fat percentage has a relatively small influence on *T_c_*, with independent influences on thermoregulatory function during exercise only with large differences (>20%) between groups or individuals (e.g., obesity). Dervis and colleagues calculated (based on another authors data [75]) that a 20% difference in body fat only elicits a ~3%–5% difference in whole-body mean specific heat, perhaps explaining the similarities between groups in some studies. Obese individuals pose a lower mean specific heat capacity compared to lean individuals, which, coupled with blunted sudomotor responses, may explain the greater *T_c_* rise [68]. Prior studies have also suggested the insulative properties of adipose tissue may attenuate heat loss at the skin [76], but this was not observed by Dervis et al. [68] and may only be relevant in cool conditions when SkBF is minimal and subcutaneous fat is of greater importance. Many of these biophysical principles may hold true in children but warrant further research to explore the independent effects of adiposity on thermoregulation, with appropriate physiological adult–child comparisons requiring workload adjustment for body mass and BSA to avoid systematic bias. 

Body composition is an important factor for consideration in outdoor physical activity guidelines for children, in addition to hydration, fitness level, acclimation status, disease, and medications, all of which can modulate thermoregulatory responses and ultimately risk of heat-related illness and injury. Although beyond the scope of this review, it is also pertinent to recognize that obesity is associated with a multitude of risk factors for the development of cardiovascular disease, including insulin resistance, dyslipidemia, and hypertension. Obesity is a proinflammatory condition that contributes to the pathogenesis of obesity-linked diseases, including metabolic dysfunction, cardiovascular diseases, and associated comorbidities [77]. In light of the growing incidence of childhood obesity [62,63], associated comorbidities [78], and increased polypharmacy at increasing younger ages [79,80], development of significant vascular dysfunction and cardiovascular disease in children and young adults is a sobering reality [78,81,82]. This has serious implications for long-term cardiometabolic risk profiles [82,83], in addition to thermoregulatory dysfunction contributing to increased heat illness risk [84,85,86,87], exacerbated by a warming global climate. 

### 4.3. Metabolic Heat Production

Children have less efficient locomotion than adults, producing more heat per unit body mass during weight-bearing activities [88,89]. Metabolic heat production can be ~10%–15% higher in children for a given workload [88], although is less pronounced during nonweight bearing activities including cycling. Studies investigating the metabolic cost of locomotion in relation to individual biomechanical variables have typically observed poor relationships. Rather, economy appears related to results from the combined effects of a multitude of factors [90]. Frost and colleagues [91] investigated metabolic, kinematic, and electromyographic responses of varying ages of children (7–8 years, 10–12 years, and 15–16 years) to 4 minute bouts of treadmill walking and running at increasing speeds. The best predictor of both VO_2_ and efficiency was age, but specific growth-related factors that may influence the metabolic cost of locomotion remain unclear [91]. Resting metabolic rate (per kg TBW) is higher in children than adults, decreasing during childhood [92,93]. Differences in total mechanical work, stride length [92], knee joint range of motion, gait efficiency [94], and higher respiratory frequency (and a higher cost of ventilation) may all contribute to a greater submaximal VO_2_ in children versus adults [92,93].

For children with disabilities or disease that affects mobility, low locomotion efficiency results in large energy expenditure compared to healthy or typically developing children. For example, cerebral palsy, the most common childhood disability in the United States, causes a high metabolic demand on daily activates. Walking efficiency is three times higher in cerebral palsy children, affecting participation in daily activities and overall quality of life [94]. Specific metabolic demands of activities in children are important in the context of safe exercise practices and thermoregulation in extreme environments, with important consideration and modifications to activities for children with disabilities affecting movement.

### 4.4. Cardiovascular Responses 

In comparison to adults, children produce a lower Q during similar absolute submaximal workloads [30,95,96]. Several studies have reported similar Q during exercise in children and adults, but an explanation for these discrepancies is not clear [97,98]. Lower Q in children results from a lower stroke volume (SV), attributed to their smaller body size and therefore smaller heart (left ventricle) compared to adults [95]. Partial compensation is provided by a higher heart rate, with more complete compensation resulting from a greater arterial–venous O_2_ difference, ultimately providing similar adult–child VO_2_ values despite a lower Q. Children therefore have an appropriate cardiovascular response for their size during exercise. This is demonstrated when SV is scaled to body size, virtually eliminating child–adult SV differences [95]. Similarly, Falk and Dotan calculated cardiovascular responses at fixed relative workloads corrected to provide a body mass specific Q (based on the data of Turley and Wilmore [95]), with values that were in fact ~10% higher in children than adults. 

Despite the lower, albeit size-appropriate exercise Q in children, differences in cardiovascular responses associated with thermoregulatory challenges become apparent. Children possess a greater (~20%) BSA compared to adults, shunting a greater proportion of Q to the skin to maximize dry heat losses [31,33,35]. Higher SkBF responses are evident during exercise (50% VO_2max_) in hot–dry conditions [55] and for similar rectal temperature (T_re_) responses [31]. Notably, comparisons of cardiovascular responses to heat are typically standardized to metabolic load (relative intensity). If absolute workloads were utilized, group differences would be artificially exaggerated owing to factors including differing locomotion and therefore greater physiological strain in children, differences in body mass and size, and potentially greater SkBF responses for dissipation of additional heat [19]. The high SkBF response declines with increasing maturational development throughout adolescence. Falk and colleagues [55] observed progressively lower SkBF responses in pre-, mid-, and late pubertal boys (significant between all groups) during cycling at 50% VO_2max_ in a hot–dry environment (42 °C, 20% rh). Considering the proportionally higher SkBF responses in children, declines in performance are understandable. Venous return and thus Q are compromised, coupled with relatively lower muscle blood flow, presenting competing demands on the cardiovascular system during exercise in the heat [99]. If conditions (heat and exercise intensity) are sufficiently extreme and exposure continues, particularly without adequate rehydration, SkBF and sweating responses may decline, compromising thermoregulatory function with potential development of heat illness [100].

### 4.5. Thermoregulatory Sweating

Many authors have reported lower local and whole-body SRs in children compared to adults [31,32,33,34]. This is typically true regardless of normalization for BSA or per sweat gland, exercise intensity, environmental conditions, and T_re_ [55,56,57]. Further, the significantly lower SRs in children appear more pronounced in comparison to adults when exposed to greater environmental extremes, higher workloads [20] or during hypohydration [101]. This leads to the widespread view that children possess inferior thermoregulatory capabilities, a notion that was challenged by Falk and Dotan [37]. Despite lower whole-body SRs, *T_c_* responses are often similar when workloads are adjusted or during passive heating, leading some to suggest that children may actually possess greater thermoregulatory efficiency during exercise and/or exposure to mild and moderate conditions [34,37]. This does not hold true in extreme ambient conditions, with children displaying greater physiological strain and increased heat illness risk.

The main focus on thermoregulatory sweating in children is typically whole-body sweating, with a paucity of data regarding RSR. Regional SRs are widely recognized in adults [102,103,104,105,106], with differences in children depending on maturational stage [34]. This was observed by Shibasaki and colleagues [34] following passive heating of prepubertal boys (7–11 years) and young men (21–25 years) via lower leg immersion in a 42 °C water bath for 60 min. RSRs varied, with some sites significantly higher than adults (forearm), some significantly lower (chest, thigh during latter 30 minutes only), and others showing no differences (back) between groups. The sweating threshold (*T_b_*) was slightly higher in boys, albeit nonsignificant (*p* < 0.10), regardless of body region. Heat-activated sweat gland density (HASG) was higher in boys at all sites, but this is not surprising given their developmental stage and smaller body size and surface area. The authors suggest regional variation in sweating associated with maturational stage and argue a peripheral ‘underdevelopment’ at the level of the sweat gland versus central drive [34]. This highlights the necessity for measuring RSR at multiple sites and the need for more detailed regional sweating data in children and adolescents at varying maturational stages. Notably, ambient conditions were 25 °C, 45% rh, providing a favorable temperature gradient for dry heat losses on which children typically rely more heavily. The authors focus on sweating in relation to maturational age, but other heat loss mechanisms should be considered. Despite lower RSR, change in *T_re_* did not differ between groups, indicating that prepubertal boys could adequately thermoregulate under mild to moderate passive heat stress in the specified conditions, relying on differing mechanisms and perhaps displaying greater efficiency.

Greater evaporative efficiency can also be attributed to the lower sweat electrolyte concentration in children [107] and serves to conserve body fluids. Children typically have higher *T_sk_*, and thus higher sweat temperatures, resulting in higher water partial pressure. The result is increased water vapor pressure and higher sweat evaporation [37]. A greater evaporative efficiency and the ability to conserve water may actually serve as an argument for thermoregulatory superiority in children versus adults in mild and moderate conditions. Maximal SRs in children have never been established for ethical reasons, but it is reasonable to assume that in the context of global climate change and increasing heat waves, children may be unable to achieve sufficient sweating rates for heat balance (maximal evaporation < required evaporation) more frequently. Maximal SRs may be improved with heat acclimation, but in uncompensable conditions, children will be unable to thermoregulate adequately, with the potential for progression to developing heat illness if precautionary measures are not employed.

### 4.6. Heat Acclimation

Heat acclimation (HA) involves a series of beneficial physiological adaptations resulting from repeated exposure to heat stress that facilitate a greater ability to deal with subsequent heat exposure. Considering children are vulnerable to heat illness under extreme conditions, employing HA strategies provides a feasible risk reduction strategy in the face of climate change. Differing HA regimens may be utilized, with conditions closest to the anticipated exposure environment, typically lasting 7–14 days [108,109,110,111] and often incorporating exercise to elicit a maximally heat acclimated phenotype [112,113]. Physiological adaptations to HA have been extensively documented in adults [104,114,115,116,117,118,119,120], including reduced cardiovascular strain (heart rate (HR) and Q) at a given workload, a lower *T_c_* threshold for sweating, increased thermosensitivity resulting in a higher SR for a given *T_c_*, and greater maximal SRs [111,116,119,121,122,123,124,125]. Children acclimate similarly to adults yet do so more slowly, requiring several more days of exposure for complete HA [72]. The reduced sweating onset threshold and HA-associated increases in SR are highly beneficial during subsequent exposure to high ambient temperatures in a population that has significantly lower sweating responses compared to adults. Children display lower sweat NaCl concentrations compared to adults [107], with reductions in sweat Na^+^ and Cl^−^ concentrations in both groups following HA [126,127]. Further acclimation increases the responsiveness of the duct to aldosterone in adults [128], but this mechanism has not been investigated in children.

A pertinent consideration with increasing prevalence of childhood obesity (Section 2.4. Body Composition) is that obese children appear to possess a lower level of acclimation versus lean boys, likely resulting from the partial acclimation acquired through training. Obese boys show higher resting and exercise *T_c_*, elevated HR responses, and lower SRs, indicating higher physiological strain [72]. Sedentary, obese children may therefore benefit most from carefully monitored HA regimens prior to summer months. In the instance of athletes, similarly to adults, exercise intensity should be progressively increased to emulate competition. A gradual increase in the number of training sessions per day and progressive introduction of protective clothing that may hinder heat loss should be adopted [129]. Considering the tendency for children to voluntarily dehydrate, special attention should be given to rehydration strategies during HA to limit hypohydration and maximize cardiovascular adaptations [130].

Ultimately, HA reduces cardiovascular strain, improves performance, and increases survivability to extreme environmental conditions, making this a critical component in reducing heat-related illness and injury in the pediatric population [72,111,116,119,121,122,123,124,125,129]. Most of the evidence available indicates similar HA responses between children and adults, but further work is warranted if HA regimens become widely adopted as ambient temperatures continue to increase, with additional consideration for body composition, disease conditions, and medication use.

### 4.7. Sweat Composition and Fluid Intake 

Fluid intake is of considerable importance in relation to thermoregulatory function, with hypohydration considered a major risk factor in the development of heat illness [100,131,132,133,134,135]. In adults, even mild hypohydration (<2% body weight) impacts cardiovascular and sudomotor responses, reducing SkBF [136,137], delaying both the vasodilatory and sweating onset thresholds, and reducing sweating rates for a given *T_c_* (reduced gain) [137,138], attenuating thermoregulatory function and heat tolerance [137]. For extensive reviews regarding the physiology of water balance, hydration assessment, strategies, and pathology, please refer to other reviews within this Special Issue. In the context of this review, hypohydration in children with consideration for rising global temperatures and concerns over predicted water shortages accompanying climate change are addressed.

In the pediatric population, voluntary dehydration is common, largely owing to children feeling a limited need to replenish fluids even when intake is insufficient [139]. This increases susceptibility to hypohydration and subsequent physiological and psychological impairments. Fluid intake and euhydration are essential for physiological function and health, in addition to cognitive function and mood in both children [140,141] and adults [142]. Hypohydration attenuates SRs in both populations, with notable thermoregulatory impact on children due to their already lower sweating responses for a given workload. Children experience a greater rise in *T_c_* for a given level of hypohydration versus their adult counterparts, increasing physiological strain and thus reducing tolerance to the heat. Hypoydration is recognized as a major risk factor for heat-related illness and injury, including progression to life threating heat stroke [52,101].

Both adults [143] and children [139] often hydrate inadequately, but the effects of chronic low fluid intake and dehydration on health are not well understood. As global climate change progresses, knowledge of the complex and multifaceted interplay of factors influencing thermoregulatory function is important. Although speculative, consideration needs to be given to pediatric fluid intake as it pertains to chronic health, with the mindset of encouraging “preventative” habits for long-term health and risk reduction. In adults, fluid intake (typically self-reported) provides conflicting results regarding cardiometabolic disease risk, in part due to failure of investigators to report plain water consumption versus intake of other fluids, including sweetened beverages [144]. Evidence does indicate low fluid intake is independently associated with developing hyperglycemia [145], type 2 diabetes mellitus [146], and renal dysfunction including chronic kidney disease (CKD) [144], with inconsistent evidence of cardiovascular disease risk. The putative link between hydration and long-term health in adults remains controversial but certainly warrants consideration in children. This is particularly concerning in light of climate change potentially exacerbating dehydration risk, coupled with the high incidence of overweight and obese children in the western world, already at increased risk of cardiometabolic disease. Limited research has assessed the relationship between fluid intake and childhood obesity, but there is some indication that obese children are less hydrated compared to those of normal weight. Maffeis and colleagues [144] determined significantly lower hydration in obese versus normal weight children (7–11 years), based on average free water reserve over 48 h. In adults, Guelinckx and colleagues [144] raise the concept of increased plain water intake as a mechanism to reduce renal and metabolic dysfunction. Risk reduction for CKD and vascular dysfunction in adults has also been observed with high water intake (>2.6 L/day) but not sweetened beverages [147,148,149]. In children, albeit speculative, perhaps this approach serves to not only improve hydration and thermoregulatory function but reduce or limit the risk of renal and metabolic dysfunction.

Appropriate replacement of both electrolytes and water should be achieved during exercise and/or in warm conditions, yet limited data concerning sweat composition in children and adolescents are available, with subsequent hydration guidelines for children derived from adult data. Maintaining appropriate water balance is important, but beverage flavor and composition are of particular significance when SRs are high. During exercise in the heat, children consume significantly more fluid when beverages are flavored compared to plain water [150]. Bar Or and colleagues [150] observed that voluntary dehydration was significantly reduced when chilled water (8–10 °C) was grape-flavored versus plain during 3 h of intermittent exercise in the heat (35 °C, 40–45% rh). Further increases in fluid consumption were observed when the beverage was not only flavored but also contained carbohydrates (6%) and NaCl (18 mmol/L), resulting in mild overhydration (0.47% increase body weight). Physiological and perceptual variables, including *T_re_*, *T_sk_*, HR, thirst, and stomach fullness did not differ between conditions, which may be explained by the relatively minimal dehydration of only−0.65%, −0.32%, and+0.47% of body weight for plain water, flavored water, and flavored carbohydrate/NaCl water, respectively. Current guidelines by The American Association of Pediatricians [52] recommend consumption of cooled, flavored beverages to mitigate dehydration in the heat, with the addition of 15–20 mmol/L NaCl increasing ad libitum drinking by up to 90% versus plain water. Sweat composition and optimal composition of fluid-replacement beverages has been extensively studied in adults [150,151,152,153,154], but limited data are available in children, with an emphasis on pathophysiology, specifically cystic fibrosis. 

There are many individual factors that can influence sweat electrolyte losses and therefore replacement, including age, physical fitness [155,156], and acclimation status [126,157]. Sweat electrolyte concentrations are a function of SR [158], with higher sweat Na^+^ and Cl^−^ concentrations evident at higher SRs resulting from reduced reabsorption in the sweat duct [157,159]. Wide variation in sweat lactate concentrations has been reported, with data indicating an inverse correlation with SR [160,161], positive correlation [162], or no correlation [163]. Several studies have demonstrated significantly lower sweat lactate concentrations with maturational age (pre- versus late-pubescent boys) [164] and in children versus adults [160] during the initial stages of moderate exercise in the heat, but not during subsequent bouts. Despite varying data concerning the relationship between sweat lactate and SR, it has been proposed that sweat lactate is higher in children owing to their lower SR compared to adults. However, sweat lactate tends to decrease with increasing exercise duration in the heat [160], and the similarity in child and adult sweat lactate during latter stages of exercise cannot be explained by SR. One proposed mechanism is the reliance on anaerobic metabolism in a sweat gland during the initial stages of sweating, and an increasing reliance on oxidative phosphorylation as exercise progresses [160]. Regardless of the mechanism, identifying sweat electrolyte losses and adapting hydration strategies is vital.

Similarly to lactate, sweat NH_3_ is significantly higher during initial stages of exercise than adults, which is thought to prevent further decreases in sweat pH through protonation of NH_3_ to NH_4_^+^. This is also reflected in the lower sweat pH observed in children during the initial stages of moderate exercise in the heat (≤20 min) [160]. Further, an inverse correlation has been observed between sweat H^+^ and Na^+^, thought to relate to acidification of sweat via tubular antiporters reabsorbing HCO^−^ and/or secreting H^+^ in exchange for Na^+^ reabsorption, potentially explaining the greater Na^+^ reabsorption in children [160].

Understanding optimal fluid composition and replacement strategies should be a priority in children, not only for performance but also overall health and safety. Hydration guidelines in children are often based on adult data, yet adult–child differences in sweat electrolyte concentrations, variation with intensity and duration, and marked differences in SR are evident. Coupled with greater voluntary dehydration in children [101], beverage composition and hydration guidelines need tailoring specifically to children and the intensity and environment in which they are exercising. Drink palatability, including flavor and temperature, are also important when considering fluid replacement in children. In the face of predicted rising average global temperatures, increased severity and frequency of heatwaves, and predicted water shortages, tailored hydration strategies will be increasingly important not only for those exercising in the heat, but for daily activates and prolonged periods outside in the heat (i.e., summer camps). 

## 5. Emerging Environmental Challenges: Effects on Thermoregulation and Health

The World Health Organization (WHO) first published an atlas outlining global environmental challenges to children’s health in 2004, which has since been expanded and updated to highlight emerging environmental stressors and strategies for exposure reduction [165]. Rising global temperatures and heat waves are only one component of environmental hazards facing children, with exposure to multiple physiological and psychological stressors influencing growth, development, and long-term health. In the context of thermoregulation, stressors including pollution, UV exposure, and water shortages may not only directly affect physiological function, but a complex interplay of multiple stressors may plausibly modulate thermoregulatory function in children (e.g., via impacts on the cardiovascular system), potentially increasing risk associated with heat-related illness and associated complications. Currently, little is known about the effects of multiple stressors on thermoregulatory function in children. 

### 5.1. Pollution 

Acute exposure to pollutants and toxic substances can impair thermoregulatory responses [166], with chronic exposure resulting in a multitude of long-term health complications. Children are reported to spend significantly greater periods of time outdoors compared to adults, potentially increasing their likelihood for exposure to toxicants. It is widely recognized that high ambient temperatures increase the uptake of many pollutants and play a critical role in increasing toxicity [167], which may be further compounded by exercise in the heat [167]. Pollutants and toxins that alter metabolism, SkBF, and/or sweating responses may have a profound effect upon thermoregulation responses of both children and adults [168]. In rodents, an acute, protective hypothermia followed by a rebound, sustained hyperthermia is observed following dosing with many toxic substances, including insecticides. Humans do not typically experience the magnitude of hypothermia observed in rodents following toxicant exposure, although marked hypothermia has been observed in specific instances [169]. More common is a hyperthermic or fever response that may persist for several days after exposure to a toxic agent [167]. Importantly, *T_b_* affects both uptake and toxicity of substances, which in turn impacts *T_b_* regulation. Based on increasing levels of pollution from vehicles, insecticide use, and byproducts from industry, an understanding of routes of absorption, toxicity, modulation of physiological responses, including temperature regulation, and long-term health effects is necessary. Increasing interest in the complex interplay among pollutants, mortality, and heat stress in older individuals is evident [17,170], which should be extended to other vulnerable populations. 

The WHO estimates the global cost resulting from pollution at 1.7 million children deaths per year [171]. High ambient temperatures and airborne pollutants independently increase morbidity and mortality, but few studies have investigated the interplay between both stressors [172,173]. A limited number of studies have attempted to do so, yielding mixed results. Several studies indicate an interaction effect, with pollution effects being greater on days with higher ambient temperature [17], whilst others found limited or no interaction [172]. Toxicity of air pollutants can be modified by atmospheric transformations, with greater primary pollutants forming toxic secondary products under conditions of higher temperature, greater sunlight exposure, and in the presence of copollutants [174]. Considering predicted elevations of both stressors in the future, and limited current knowledge of how their interactions affect thermoregulation and overall health, further research is urgently needed. The physiological strain associated with exposure to high ambient temperatures may alter the physiological response to pollutants and other chemicals, increasing susceptibility to the negative health effects [172,173]. A further plausible consideration is the deleterious cardiovascular and respiratory effects of airborne pollution that may lead to compromised thermoregulatory function both in children and later in life, raising health risks in an already vulnerable population during extreme heat exposure.

An increasing number of studies have elucidated an association between residential proximity to high traffic roads and, thus, airborne vehicle pollution and a multitude of adverse health events and conditions. Impacts on the respiratory system have been widely studied [175], and increasing literature is indicating a concerning impact on the cardiovascular system.

Air pollution has been implicated as proatherogenic, increasing likelihood of cardiovascular events [176]. Vehicle emissions are a major contributor to outdoor pollution, accounting for up to 90% of pollutants, including carbon dioxide (CO_2_), carbon monoxide (CO), oxides of nitrogen (NO_x_), particulate matter (PM), ozone (O_3_), volatile organic compounds (VOCs) including benzene and formaldehyde, and other byproducts. Many of these pollutants are linked to numerous adverse health conditions and events, particularly relating to respiratory function [177] and cancer risk [178]. Specifically, PM from vehicle exhaust emissions has been linked to adverse cardiovascular events [179,180]. Of concern to climate change, higher ambient temperatures are linked to greater respiratory uptake of pollutants such as O_3_ and greater toxicity [167].

In adults, literature is varied, with some evidence that long-term exposure to urban pollution may increase arterial stiffness and carotid intima–media thickness, regardless of pre-existing Cardiovascular disease (CVD) [181]. Potential mechanisms include signaling cascades stimulating pro-inflammatory cytokine release, ROS production, endothelial dysfunction, and vascular smooth muscle remodeling, ultimately attenuating vascular function and promoting atherogenesis [182,183]. Children are at greater risk of negative health effects associated with pollution, owing to a complex interplay of factors, including immature immune responses, small lung volumes, higher respiratory rates, tendency for mouth breathing, and longer time periods spent outside [184]. Children exposed to urban air pollution show increased markers of oxidative stress, inflammation, and endothelial dysfunction [16,185]. Notably, this an understudied area of toxicology and physiology, with only one study indicating that long-term urban residency of children living in close proximity to a major road (30–300 m) was linked to increased carotid arterial stiffness versus those living further away.

Armijos and colleagues [186] observed that long-term residency in close proximity to a high traffic road (<100 m), and thus airborne pollution, stimulates arterial remodeling (carotid intima–media thickness) in children aged 7–12 years versus those living further away (>200 m), with control for covariates and risk factors for atherosclerosis [186]. Many factors can influence inflammation and CVD risk, but there is a need for long-term, longitudinal, epidemiological studies to determine the cumulative effects of pollution on cardiovascular health, with putative implications for thermoregulatory function and exercise capacity. Exposure to proatherogenic environmental factors may cause early progression to clinical disease, rendering current knowledge of vascular responses in children inaccurate with the potential for overestimating heat loss potential in certain conditions. Whilst currently speculative, elucidating if pollution has functional consequences for thermoregulation in children is vital in the face of multifaceted physiological stressors, including climate change and increasing prevalence of noncommunicable diseases.

### 5.2. Ultraviolet Exposure 

Exposure to UV radiation can have beneficial or detrimental effects depending on a complex interplay of factors, including the wavelength of UV radiation, extent of exposure, skin pigmentation, and other individual factors [2]. Depletion of protective stratospheric ozone has led to increased UVB (315–400 nm) levels reaching the earth’s surface [187] and is therefore of concern to human health. The epidermis absorbs the majority of UV that irradiates the skin, with only longer wavelengths transmitted deeper into the dermis. UVB has therefore been the predominant focus of research, but interest is growing in the effects of the UVA waveband (315–400 nm). Environmental change coupled with lifestyle choices in recent decades, including increased leisure time, popularity of beach/sunshine vacations, poor sunscreen habits, use of tanning beds, and fewer clothes worn outdoors, places individuals at risk of irradiation. This has pertinence to both adults and children; however, some evidence indicates that UV radiation exposure during childhood may be of greater importance for both beneficial and detrimental effects compared to exposure later in life [188].

Despite the largely negative literature surrounding UV exposure, it is important in many physiological processes, with skeletal disease and vitamin D insufficiency associated with inadequate exposure. Some evidence indicates a regulatory role in blood pressure via calcium homeostasis and vitamin D_3_, with artificial UVB exposure lowering blood pressure in mild, unmedicated hypertensives [189]. Photoimmunology, an emerging field of study regarding how UV radiation impacts immune function, is a reminder of how limited current knowledge is on topics such as UV and effects on many physiological processes. UV radiation is typically immunosuppressive, yet increasing evidence suggests a protective role of UV radiation in autoimmune diseases, including Type 1 diabetes, rheumatoid arthritis, and multiple sclerosis [14]. This occurs via multiple signaling mechanisms, including local and systemic immunosuppression, the immunomodulatory effects of vitamin D (1,25-dihydroxycholecalciferol), and suppression of melatonin secretion and subsequent T cell responses [190,191,192,193]. Overall, it appears that UV radiation exerts immunomodulatory effects, primarily via suppression of helper T cell type-1 mediated responses thorough multiple mechanisms. This may be beneficial in specific circumstances, including autoimmune diseases, but requires further investigation in humans.

Excessive exposure to UV radiation is associated with many negative health effects, including increased skin cancer risk [13,194,195], eye damage, and suppressed immune function [196], with further deleterious health effects becoming more apparent. In instances of sunburn, skin injury results from penetration of UVB into the dermal and epidermal layers, causing damage to cutaneous blood vessels and eccrine sweat glands [197]. Mild artificial sunburn appears to attenuate SRs and sensitivity for at least 24 h, with potential for impaired thermoregulatory function if this occurs over a large body surface area [197]. Greater time spent outdoors by children compared to adults, poor sunscreen habits, and increasing UVB levels increase the likelihood of sunburn in a pediatric population, with potential for thermoregulatory dysfunction. Specific dermal conditions have been shown to impair thermoregulation and reduce heat tolerance [198]. Evidence is currently limited with regard to sunburn and thermal tolerance, but further studies examining the acute and chronic effects on eccrine sweat gland function and vascular responses are necessary in light of changing environmental conditions. Evidence of UVB-induced alterations in cutaneous vasodilation have recently been reported in adults but, as an important component of the thermoregulatory response, may have relevance for thermoregulation in both children and adults. Children rely more heavily on cutaneous vasodilation and ‘dry’ heat losses for thermoregulation compared to adults, making decrements in this response potentially more problematic. Nitric oxide (NO) is a potent vasodilator, produced from the substrate L-arginine via nitric oxide synthase (NOS) isoforms, and is necessary for full expression of the cutaneous vascular response to both local [199,200,201] and whole-body heating [202]. Recent work by Wolf et al. [203,204] indicates deleterious effects of acute UV radiation exposure on cutaneous microvascular function. Specifically, an acute dose of UVB radiation (300 mJ.cm^2^, 75 s) was shown to attenuate NO-dependent vasodilation on the ventral forearm, likely via its putative degradation of 5-methyltetrahydrofolate (5-MTHF) and subsequent reactive oxygen species (ROS) signaling [203]. A further study by this group [204] indicated attenuated NO-dependent skin blood flow responses to a local heating protocol (42 °C) following acute broad spectrum UV radiation exposure (450 mJ.cm^−2^, 75 s) versus control (non-exposure). UV radiation-mediated reductions in NO-dependent vasodilation were prevented with application of SPF 50 sunscreen or ‘simulated sweat’. Notably, this was conducted under very acute conditions (60–75 s) in a young, healthy adult population, but consideration must be given to implications in children and adolescents. Children often spend greater time outdoors than adults, and rely more heavily on dry heat losses, shunting a greater proportion of Q to the skin during passive and exercise heat exposure. Whether long-term effects of chronic, repeated UV exposure on cutaneous vascular reactivity translate to meaningful physiological outcomes and compromised thermoregulatory function is currently unknown. As average global temperatures rise and broad spectrum UV radiation exposure increases, in part due to decreasing atmospheric ozone [187], understanding if these factors modulate cutaneous vascular responses, and thus thermoregulation, is certainly warranted.

## 6. Child Health, Safety, and Risk Reduction

Based on global climate predictions, awareness of the factors increasing risk of heat-illness in children is important in prevention, with particular emphasis on dehydration, current or recent illness, chronic conditions, and specific medications [52]. In particular, illness involving gastrointestinal distress (particularly vomiting and diarrhea), and conditions and medications that affect thermoregulation, exercise tolerance, and water–electrolyte balance should be addressed. Common examples include type 2 diabetes [205,206,207], obesity [72,208], cystic fibrosis [209], diabetes insipidus, anticholinergic medications, diuretics, dopamine [210], and serotonin uptake inhibitors.

A second important step in the prevention of heat-related illness and injury is understanding and implementing HA. Children acclimate to heat more slowly than adults [72] but will beneficially increase sweat capacity, reduce the onset threshold for sweating, and reduce overall physiological strain [72,208]. Obese children possess lower levels of acclimation and therefore should be acclimated more progressively during summer months [72]. When exercising in the heat, children should follow appropriate hydration and exposure guidelines for the ambient conditions, with special consideration and awareness of their propensity for greater voluntary dehydration [101]. Hypohydration compromises thermoregulatory function to a greater extent in children compared to adults, making it an import factor to mitigate health risks during exposure. The American Academy of Pediatrics position stand addressing “Climatic Heat Stress and Exercising Children and Adolescents” [52] recommends multiple risk reduction strategies, including but not limited to:

1. Increasing rest periods. Activities lasting >15 min should be reduced in conditions of high solar radiation, high humidity, and ambient temperatures above critical limits.
2. When beginning a strenuous exercise program or travelling to a warmer climate, HA over 10–14 days should be planned with reduced exercise intensity, duration, and protective clothing.
3. Ensure adequate hydration prior to extended exercise in the heat. Intermittent drinking periods should be enforced, regardless of thirst (100–250 mL every 20 min). Weighing a child pre/post-exercise can assist with verifying hydration.
4. Lightweight, light-colored, single-layer clothing should be worn that is absorbent. Sweat-soaked garments should be replaced.
5. Child education on heat illness and hydration practices should be adopted to help raise awareness of prevention, and recognition of the signs and symptoms of heat-related illness and injury. Trained staff should be present, and an emergency plan should be in place.

Considering the increasing incidence of a multitude of childhood diseases, awareness of diseases that alter thermoregulatory responses, recent illness, specific medications, and mental retardation (for example, a compromised ability to recognize dangers of heat, not rehydrating) are also important. Education surrounding behavioral thermoregulation is vital, including seeking shade, requesting help when needed, and using cool water and damp towels to help to reduce body temperature during hyperthermia. Sunscreen should be applied to reduce acute effects of UV radiation on cutaneous vascular responses in the heat and avoid sunburn. Clothing insulation and requirements in children in varying environmental conditions are beyond the scope of this review but are an important consideration. Readers are directed to other excellent reviews [211]. Emerging evidence highlights the complex interplay between multiple stressors, modulating physiological responses, and, therefore, morbidity and mortality risk. Exposure to increasing global temperatures necessitates consideration of heat stress in cumulative risk assessment [43], due to potential temperature-related modulation of toxicity of air pollution and other abundant chemicals, for example, pesticides [174], exacerbating health effects. Children are exposed to many physical and chemical stressors, including heat, UV radiation, sunlight, noise, infection, psychosocial stressors, and pollution. Long-term exposure may facilitate earlier progression to clinical disease, altered physiological responses versus those observed in healthy children, and ultimately premature mortality. The long-term impact of multiple environmental–physiological factors on physiological function are currently not fully understood. Combined efforts between thermal physiologists, epidemiologists, climate physiologists, and regulatory bodies/policymakers are necessary to produce more comprehensive heat protection policies, incorporating technology, clothing, and behavioral adaptations as global climate change advances and limits of human tolerance are reached [45].

## 7. Summary

Understanding physiological responses to heat exposure is of increasing importance in the face of extraordinary environmental and climate change facing humans. In particular, emphasis should be placed on ‘at risk populations’, including children and infants, the elderly, and individuals with obesity and chronic pathologies, including cardiovascular, renal, and metabolic diseases. Many physiological differences exist between adults and children of varying developmental stages in their responses to exercise and exposure to environmental extremes, including morphological, endocrine, cardiovascular, metabolic, and thermoregulatory responses. The classic notion that children possess inferior thermoregulatory capabilities and reduced thermal tolerance has been vigorously challenged over the past 25 years and may only hold true in extreme conditions. Of course, this is particularly relevant in light of global climate change where ‘extremes’ are more likely to be encountered, evidenced by patterns of increasing average global ambient temperatures, increased frequency and severity of heat waves, and increased pollution and UV exposure. The complex interplay between increasing environmental stressors should be carefully considered when coupled with concerning health statistics that negatively affect thermoregulation in children, including prevalence of inactivity, childhood obesity, and the earlier onset of preventable ‘adult’ diseases, including type 2 diabetes and dyslipidemia. The long-term impact of multiple environmental–physiological stressors, including heat, UV exposure, and pollution on physiological function in children, is not fully understood and warrants further investigation as a significant future global health challenge.
